# Determination of the Unilaterally Damaged Region May Depend on the Asymmetry of Carotid Blood Flow Velocity in Hemiparkinsonian Monkey: A Pilot Study

**DOI:** 10.1155/2022/4382145

**Published:** 2022-11-09

**Authors:** Jincheol Seo, Kyung Seob Lim, Chang-Yeop Jeon, SeungHo Baek, Hyeon-Gu Yeo, Won Seok Choi, Sung-Hyun Park, Kang Jin Jeong, Jinyoung Won, Keonwoo Kim, Junghyung Park, Jiyeon Cho, Jung Bae Seong, Minji Kim, Yu Gyeong Kim, Jae-Won Huh, Samhwan Kim, Yong Hoon Lim, Hyung Woo Park, Hye Min Tak, Man Seong Heo, Ji-Woong Choi, Sun Ha Paek, Youngjeon Lee

**Affiliations:** ^1^National Primate Research Center, Korea Research Institute of Bioscience and Biotechnology (KRIBB), Daejeon 28116, Republic of Korea; ^2^Futuristic Animal Resource and Research Center, KRIBB, Daejeon 28116, Republic of Korea; ^3^Department of Functional Genomics, KRIBB School of Bioscience, Korea University of Science and Technology, Daejeon 34113, Republic of Korea; ^4^School of Life Sciences, BK21 Plus KNU Creative BioResearch Group, Kyungpook National University, Daegu 41566, Republic of Korea; ^5^Brain Engineering Convergence Research Center, Daegu Gyeongbuk Institute of Science and Technology, Daegu 42988, Republic of Korea; ^6^Department of Electrical Engineering and Computer Science, Daegu Gyeongbuk Institute of Science and Technology, Daegu 42988, Republic of Korea; ^7^Movement Disorder Center, Department of Neurosurgery, Seoul National University Hospital, Seoul 03080, Republic of Korea; ^8^Department of Neurosurgery, Clinical Research Institute, Seoul National University Hospital, Hypoxia/Ischemia Disease Institute, Cancer Research Institute, Seoul National University College of Medicine, Seoul 03080, Republic of Korea; ^9^Advanced Institutes of Convergence Technology (AICT), Seoul National University, Seoul 16229, Republic of Korea; ^10^Department of Information and Communication Engineering, Daegu Gyeongbuk Institute of Science and Technology, Daegu 42988, Republic of Korea

## Abstract

The hemiparkinsonian nonhuman primate model induced by unilateral injection of 1-methyl-4-phenyl-1,2,3,6-tetrahydropyridine (MPTP) into the carotid artery is used to study Parkinson's disease. However, there have been no studies that the contralateral distribution of MPTP via the cerebral collateral circulation is provided by both the circle of Willis (CoW) and connections of the carotid artery. To investigate whether MPTP-induced unilaterally damaged regions were determined by asymmetrical cerebral blood flow, the differential asymmetric damage of striatal subregions, and examined structural asymmetries in a circle of Willis, and blood flow velocity of the common carotid artery were observed in three monkeys that were infused with MPTP through the left internal carotid artery. Lower flow velocity in the ipsilateral common carotid artery and a higher ratio of ipsilateral middle cerebral artery diameter to anterior cerebral artery diameter resulted in unilateral damage. Additionally, the unilateral damaged monkey observed the apomorphine-induced contralateral rotation behavior and the temporary increase of plasma RANTES. Contrastively, higher flow velocity in the ipsilateral common carotid artery was observed in the bilateral damaged monkey. It is suggested that asymmetry of blood flow velocity and structural asymmetry of the circle of Willis should be taken into consideration when establishing more efficient hemiparkinsonian nonhuman primate models.

## 1. Introduction

Hemiparkinsonian nonhuman primate (NHP) models induced by unilateral injection of 1-methyl-4-phenyl-1,2,3,6-tetrahydropyridine (MPTP) into the carotid artery are widely used to investigate the early stages of Parkinson's disease (PD) [1–6]. The approach of using the internal carotid artery as an MPTP delivery route based on the cerebrovascular anatomical structure has several advantages over intramuscular administration. First, a NHP-PD model with PD symptoms can be established, even with a single injection of MPTP. Second, this animal model can induce unilateral damage to dopaminergic neurons. However, there have been no studies that the contralateral distribution of MPTP via the cerebral collateral circulation is provided by both the circle of Willis (CoW) and connections of the carotid artery [7, 8]. Cerebral collateral blood flow helps to maintain adequate oxygenation, supports cellular function in both hemispheres, and plays a protective role in patients with stenosis or occlusion in one of the cerebral arteries [9–12]. The CoW is considered a collateral flow route. And the redistribution of cerebral blood is affected by the asymmetry in blood flow in both carotid arteries that may be induced by stenosis or occlusion [11, 13, 14]. In this study, we aimed to investigate whether the laterality of MPTP-induced damage was underpinned by a relative asymmetry of cerebral collateral blood flow [15, 16]. Three monkeys were infused with MPTP via the left internal carotid artery (ICA) using an interventional radiology method that permits minimally invasive delivery of MPTP. To evaluate PD progression, we analyzed changes in parkinsonian behavior scores, global activity, apomorphine-induced rotation behavior, plasma Regulated on Activation, Normal T Cell Expressed and Secreted (RANTES) levels, and positron emission tomography (PET) imaging of dopamine transporters (DATs) using ^18^F-FP-CIT before/after left ICA infusion of MPTP. To compare asymmetric damage of striatal subregions, striatal asymmetry indexes (SAIs) were calculated based on ^18^F-FP-CIT binding potential (FP-CIT BP) results of five monkeys that received intramuscular (IM) MPTP injections in our previous study [17]. The five monkeys in the previous study showed low asymmetry (<0.5 SAIs) in FP-CIT BP after repeated IM injections of MPTP. This result suggested that repeated IM injections of MPTP-induced bilateral symmetric striatal damage. To examine correlations between various damaged regions (unilateral or bilateral) in basal ganglia and cerebral blood flow, including structural asymmetry in the CoW and common carotid artery (CCA) blood flow velocity (BFV), Bilateral CCA BFV was quantified using quantitative flow (Qflow) measurements performed using 2D-phase contrast angiography (PCA) techniques [18]. Further, we investigated the relationship between the asymmetry of an anterior cerebral artery (ACA) A1 segments in the CoW with cerebral blood flow lateralization and collateral compensatory mechanisms [19, 20].

## 2. Materials and Methods

### 2.1. Experimental Animals and Ethics Statement

Three female cynomolgus monkeys (*Macacafascicularis*, C1, C2, and C3) were used in this study. All monkeys were 12 years old with a body weight ranging between 2.7 and 3.3 kg. All NHP experiments in this study were approved by the Korea Research Institute of Bioscience and Biotechnology Institutional Animal Care and Use Committee (Approval No. KRIBB-AEC-20290) and have been reported in compliance with the ARRIVE guidelines [21, 22]. As previously reported [17, 23–27], all experimental monkeys were used that were maintained at the National Primate Research Center at the Korea Research Institute of Bioscience and Biotechnology (KRIBB). To prevent potential damage to other monkeys via the metabolites of MPTP, monkeys were averted from having physical contact but were allowed visual and auditory contact with their neighbors. Cage sizes were 60 cm × 80 cm × 80 cm and met the guidelines of the National Institutes of Health (USA). Commercial monkey chow (Teklad 2050™, Envigo, USA), various fruits, and water (*ad libitum*) were provided and rubber and plastic toys were also supplied for environmental enrichment. Housing conditions were maintained at a temperature of 24 ± 2°C, relative humidity of 50 ± 5%, and a 12 h light/12 h dark cycle. All monkeys were monitored by the veterinarian in accordance with the guidelines of the Weatherall report on the use of NHPs in research [28, 29].

### 2.2. Angiography and Veterinary Care

The interventional radiology method employed in this study was performed as reported in our previous studies [17, 30–33]. All monkeys were anesthetized with ketamine (5 mg/kg, I. M, Yuhan, Seoul, South Korea) and maintained with isoflurane (2%, Hana Pharmacy, Hwaseong, South Korea). Body temperature, O_2_ saturation (SpO_2_), heart and respiration rate, and blood pressure were maintained normally using the homeothermic Monitoring System (Harvard Apparatus, USA) during the interventional radiology procedures [34]. The left common femoral artery was reached using the Seldinger technique. A 5 French arterial sheath (Sungwon Medical, Cheongju, Korea) had penetrated using a 5F micropuncture set (Merit Medical, South Jordan, UT). The left proximal ICA was catheterized using a 0.035 inch guide wire (Terumo, Tokyo, Japan) and a 5F catheter (Weinberg catheter, A&A Medical, Seongnam, Korea). To acquire anterior-posterior and lateral views, selective angiography was carried out using a digital angiography unit (AlluraXper FD20, Philips Medical System, the Netherlands). Heparin (500 units, I. V bolus) was administrated after installing a femoral arterial sheath, and heparinized normal saline (3,000 units in 1,000 mL) was continuously hosed down the 5F guiding catheter to avoid thromboembolic complications during the endovascular intervention. An unsuitable endovascular occluder was moved forward to the microguidewire (Synchro 14, Boston Scientific, Fremont, CA) and placed in the internal and external carotid artery division branches of the left common carotid artery. Contrast media (Iversense 240, Accuzen, Seoul, Korea) was injected through the microcatheter (Supplemental Video 1).

### 2.3. MPTP Infusion

The MPTP infusion method was performed as reported in previous studies of hemiparkinsonian NHP models [1–4, 6, 35–39]. For each injection, 20 mL of saline containing 3 mg of MPTP-HCl (Sigma–Aldrich) was infused over 15 min at a rate of 1.33 mL/min using an infusion syringe pump. After completion of infusion, 3 mL of saline was delivered, the microcatheter was carefully removed, and the left common femoral artery incision was closed.

### 2.4. Magnetic Resonance (MR) Image Acquisition

All MRI examinations were conducted using a Philips 3T Achieva scanner (Philips Medical System, Best, Netherlands) with a 32-channel head coil. Monkeys were comfortably restricted in a supine position in a custom-made bed holder during MRI scans [17, 32, 33]. Inhaled CO_2_, O_2_ saturation, pulse, respiration rate, and body temperature were continuously monitored. Quantitative flow (Qflow) measurements were performed using 2D-phase contrast angiography (PCA) techniques with data acquisition synchronized to the heart cycle [18, 40]. Flow-sensitive and flow-compensated (reference) scans were acquired via a bipolar gradient. Flow information was represented as a function of time. The results were obtained by postprocessing Qflow. CCA BFV was acquired using a Qflow sequence with the following parameters: repetition time/echo time (TR/TE) = 13/8 ms, flip angle = 10°, the field of view (FOV) = 150 × 103 mm, matrix size = 128 × 88, slice thickness = 5 mm, NEX = 1, and PC velocity = 90 cm/s in the through-plane direction. To investigate the asymmetry in flow velocity along the location of the CCA, images were scanned vertically against the direction of blood flow in three-dimensional time of flight (TOF) images. The center of slice 1 was located 3 mm below the top end of the bifurcation of the ICA and ECA. Slices 2 and 3 were measured at 3 mm and 6 mm below slice 1, respectively. Following the completion of MRI scans, monkeys were allowed to recover from anesthesia and were reverted to their home cages. Animal monitoring was daily performed by veterinarians.

### 2.5. PET Imaging Protocol

PET imaging was carried out to measure the DAT activity using a PET/computed tomography (CT) scanner (nanoScan PET-CT, Mediso Ltd., Budapest, Hungary), as reported in our previous studies [17, 26, 33, 41]. The ^18^F-FP-CIT was acquired from DuChemBio Co., Ltd., Daejeon, Korea. 185 MBq^18^F-FP-CIT in 1.5 mL saline were administrated via the saphenous vein after CT was conducted for attenuation correction. The PET imaging analysis was performed according to the protocol reported in previous studies [17, 33].

### 2.6. Evaluation of Clinical Behavior

Spontaneous behaviors in home cages were acquired using a digital video camera (HDR-CX405, Sony Corp., Japan) positioned in front of cages. Behaviors were recorded consecutively for 240 min (12 : 00–4:00 p.m.), and the global activity of three monkeys was evaluated via analysis of the video recordings using Smart 3.0 software (Panlab S. L., Barcelona, Spain), as previously reported [17, 26]. Based on the Kurlan scale, Parkinsonian behavior scores were evaluated in the monkey PD model [17, 26, 33, 42, 43]. The minimum degree is 0 (indicating a normal behavior movement), whereas the maximum degree is 29 (indicating severe Parkinsonian motor symptoms). The evaluation was carried out by three trained experimenters who were blinded to the experimental design. Apomorphine rotation test was performed to assess for contralateral turning which is indicative of unilateral dopamine depletion. This was done by injecting apomorphine (intramuscular dopamine receptor agonists, 0.1 mg/kg, Sigma–Aldrich) or saline within 28 to 33 weeks after MPTP infusion [44, 45]. After apomorphine or saline injection, the rotational behaviors were immediately recorded consecutively for 80 min to manually count the numbers of turnings.

### 2.7. Blood Preparation and Luminex Assay

Whole blood collection and Luminex assay were performed as reported in our previous study [26]. Whole plasma was analyzed to identify the level of RANTES using a MILLIPLEX® MAP NHP cytokine magnetic bead panel kit (EMD Millipore, MA, USA). All data were collected using a Luminex-100 ™ instrument (Luminex, Austin, TX, USA) and analyzed using MasterPlex QT 2010 (mIRAIbIO, Hitachi, CA, USA).

### 2.8. Asymmetry Index (AI) and Statistical Analysis

Asymmetry indexes (AIs) were calculated using the following formula: [(a − b)]/[(*a* + *b*)/2] × 100, where a and *b* represent two different sides (a, contralateral; *b*, ipsilateral), respectively, of either the FP-CIT BP data or CCA BFV [46–50]. All statistical analyses were performed using Graphpad Prism 9 (Graphpad Software, San Diego, CA, USA). CCA BFV data were analyzed by comparing both sides in each cynomolgus monkey using a paired one-tailed Student's *t*-test. Data are expressed as means ± standard deviation. *P* < 0.05 was considered statistically significant.

## 3. Results

### 3.1. Effects of Left ICA MPTP Infusion on DAT Activity

**Figure 1:**
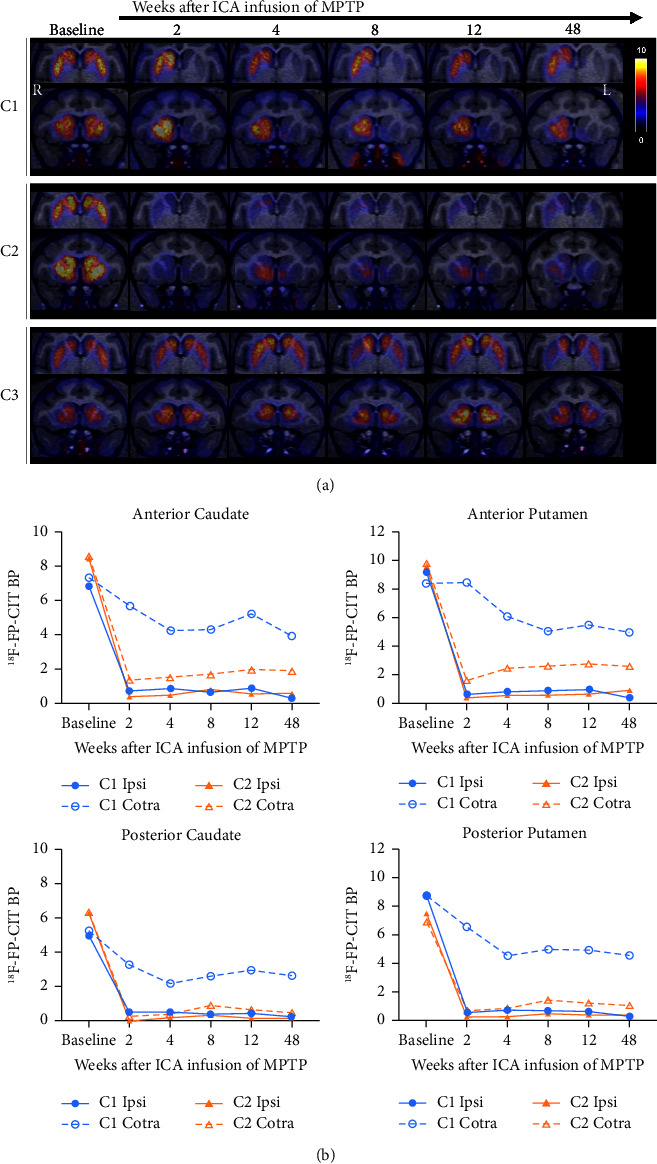
(a) Representative ^18^F-FP-CIT PET images fused with individual MRI in unilaterally damaged (C1), bilaterally damaged (C2), and undamaged (C3) cynomolgus monkeys after MPTP infusion. (b) Histogram representing ^18^F-FP-CIT binding potential in subregions of the dorsal striatum after left internal carotid artery infusion of MPTP in unilaterally (C1) and bilaterally (C2) damaged monkeys after MPTP infusion. The dotted line indicates the contralateral dorsal striatum relative to the MPTP-infused side. Binding potential, BP; Contra, Contralateral; Ipsi, Ipsilateral.

**Figure 2:**
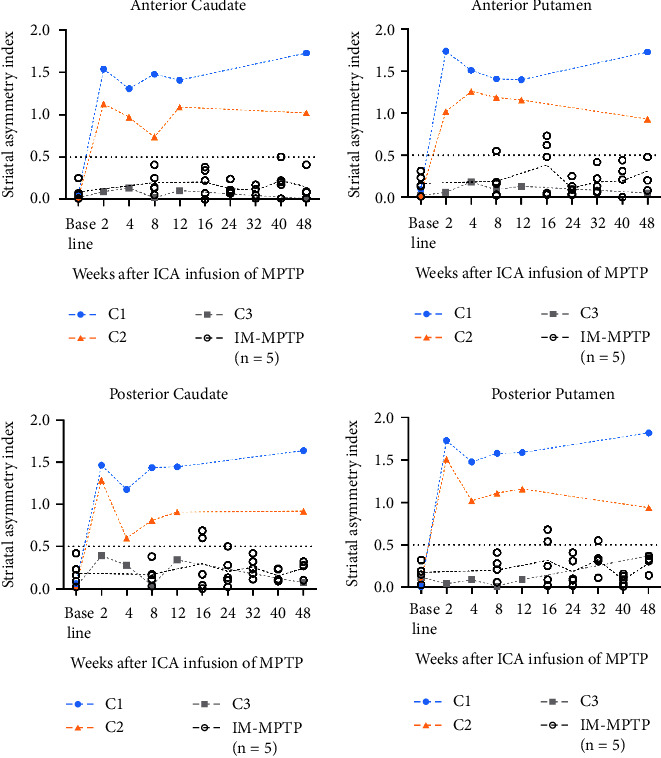
Striatal asymmetry index (SAI) was calculated using the ^18^F-FP-CIT BPs of three monkeys infused unilaterally with MPTP into the ICA in this study and five monkeys injected intramuscularly with MPTP in our previous study [17] to compare asymmetric damage of striatal subregions.

To monitor signs of PD progress, ^18^F-FP-CIT PET imaging was carried out after MPTP infusion via the left ICA at 2, 4, 8, 12, and 48 weeks following the first MPTP infusion (Figure 1). Quantitative analysis showed that ^18^F-FP-CIT BP in the striatum was reduced unilaterally in C1 monkey and bilaterally in C2 monkey but was not reduced in C3 monkey, which recovered after MPTP infusion (Figure 1(b) and Supplemental Figure 1). To compare asymmetric damage of striatal subregions, the SAIs were calculated based on absolute value calibration using the ^18^F-FP-CIT BPs of the three monkeys that received unilateral infusions of MPTP into the ICA in this study and five monkeys that received IM injections of MPTP which were used in our previous study [17] (Figure 2 and Supplemental Table 1). The SAI of IM-MPTP-injected monkeys was increased by 0.036 at 8 weeks after injection of MPTP (*n* = 5). The SAIs of unilaterally (C1) and bilaterally (C2) damaged monkeys were increased by 1.419 and 0.930, respectively. The SAI of the undamaged monkey (C3) was decreased by 0.007.

### 3.2. CoW Structure and Asymmetric Blood Flow Velocity of MPTP-Infused Cynomolgus Monkeys

**Figure 3:**
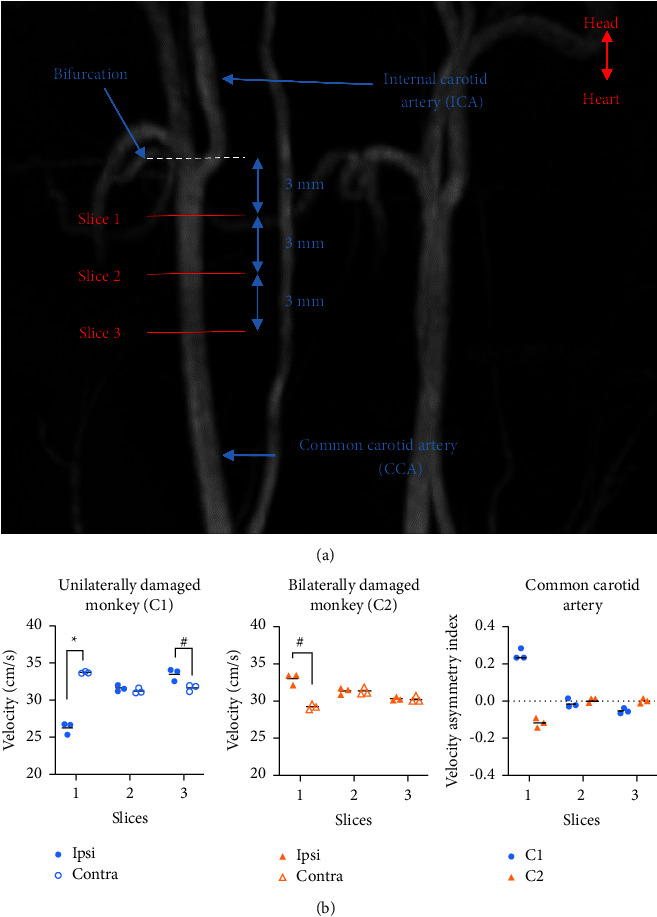
Asymmetric common carotid artery (CCA) blood flow velocity (BFV) was observed in slice 1 in the damaged region in unilaterally and bilaterally damaged cynomolgus monkeys. (a) MRA depicting the carotid artery and its bifurcation. Slice 1 was always obtained 3 mm below the wall of the far side of the bifurcation. Slices 2 and 3 were obtained 6 and 9 mm below slice 1, respectively. (b) Relatively low ipsilateral CCA BFV was observed in the unilaterally damaged monkey (C1) in slice 1 (−7.44 cm/s). In contrast, relatively high ipsilateral CCA BFV was observed in the bilaterally damaged monkey (C2) in slice 1 (+3.72 cm/s). The asymmetry of CCA BFV was calculated using the asymmetry index (AI) of velocity in slice 1 of the CCA. Student's paired *t*-test; ^*#*^*P* < 0.05, ^*∗*^*P* < 0.01.

To investigate the asymmetry of A1 segments, the diameters of ACA A1 segments, middle cerebral artery (MCA) M1 segments, ICA, and CCA were measured using MR angiography (MRA). Although symmetry of the diameters in all segments was observed in the three monkeys, the different ratio in vascular diameter of MCA to ACA of 2.0 versus 1.0 were observed in the ipsilateral side of unilaterally and bilaterally damaged monkeys, respectively (Table 1 and Supplemental Figure 2). CCA BFV was bilaterally quantified using QFlow of MRI sequences in the two damaged monkeys. Asymmetry of CCA BFV was calculated using the AI of velocity in slice 1 of the CCA. Asymmetric CCA BFV was observed in slice 1 in the damaged region in both unilaterally (0.248 ± 0.027) and bilaterally (−0.120 ± 0.026) damaged monkeys (Figure 3). In the unilaterally damaged monkey (C1), BFV in the CCA ipsilateral to MPTP infusion was lower than that in the contralateral CCA. Compared to that in the contralateral CCA, relatively low ipsilateral CCA BFV was observed in the unilaterally damaged monkey (C1) in slice 1 (Ipsi, 26.30 ± 0.77 cm/s; Contra, 33.74 ± 0.10 cm/s). In contrast, relatively high ipsilateral CCA BFV was observed in the bilaterally damaged monkey (C2) in slice 1 (Ipsi, 32.89 ± 0.76 cm/s; Contra, 29.16 ± 0.36 cm/s).

### 3.3. Effects of Left ICA MPTP Infusion on Parkinsonian Behavioral Signs and Plasma RANTES Concentrations

**Figure 4:**
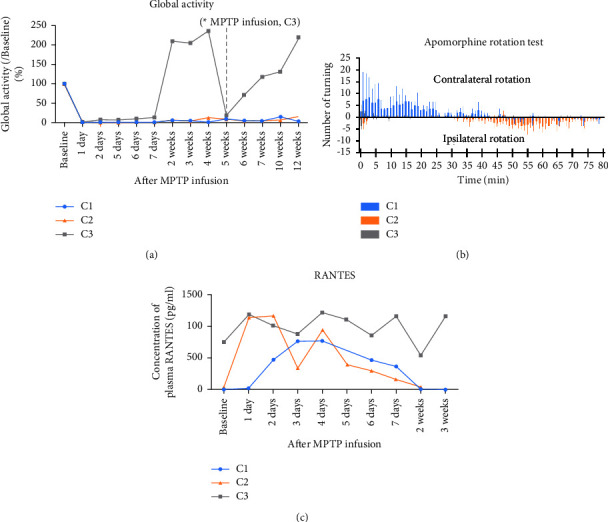
(a) Global activity was immediately reduced after left internal carotid artery infusion of MPTP in unilaterally (C1) and bilaterally (C2) damaged cynomolgus monkeys. The global activity of the undamaged cynomolgus monkey (C3) was completely recovered despite an additional infusion of MPTP. ^*∗*^Additional infusion of MPTP in the contralateral side of the undamaged cynomolgus monkey (C3). (b) Apomorphine-induced contralateral turning (positive values) in the C1 monkey (blue bar). Apomorphine rotation tests were repeated three times within 31 to 33 weeks, once a week, after MPTP infusion. The rate of contralateral turning acutely increased to 21 turns/min, and then decreased. The contralateral turning was not observed in the C2 monkey (orange bar). Plot, mean with standard deviation. (c) Plasma RANTES concentrations were increased at 1 or 2 days after MPTP infusion and normalized from 2 or 3 weeks after MPTP infusion. Plasma RANTES concentrations in the undamaged cynomolgus monkey (C3) were abnormally higher than those of the other two subjects prior to MPTP infusion; this pattern was preserved even after MPTP infusion.

Behavioral signs of PD, including parkinsonian behavior scores and global activity, were observed from days 1 to 7 following the MPTP infusion and compared to baseline levels (Figure 4(a) and Supplemental Figure 3). Two monkeys (C1 and C2) maintained parkinsonian behavioral signs at 12 weeks after infusion of MPTP. In contrast, behavioral signs of PD in one monkey (C3) recovered at 2 weeks after MPTP infusion. Despite an additional infusion of MPTP on the contralateral side, behavioral signs of PD in C3 did not persist (Figure 4(a)). Apomorphine rotation tests were repeated three times within 28 to 33 weeks, once a week, following MPTP infusion. Before the application of apomorphine (injection of saline), monkeys made ipsilateral turnings towards the MPTP infusion side at a rate of 8 turns/min (Supplemental Figure 4). After apomorphine was inoculated, the ipsilateral turning was suppressed, and turning to the contralateral side was observed in the C1 monkey (Figure 4(b)). The rate of contralateral turning acutely increased to 21 turns/min, and then decreased. Contralateral turning was not observed in the C2 monkey. Plasma concentrations of RANTES were monitored daily after MPTP infusion (Figure 4(c)). In two damaged monkeys, plasma RANTES concentrations were immediately increased after MPTP infusion (C1, 1 day; C2, 2 days after MPTP infusion, respectively) and normalized 2 weeks after MPTP infusion to preinfusion level (baseline). Plasma RANTES concentrations were higher in the undamaged monkey (C3) than in the other two monkeys prior to MPTP infusion; this pattern was maintained even after MPTP infusion.

## 4. Discussion

This is the first study to demonstrate that the level of asymmetry in the damaged region after infusion of MPTP via the ICA may depend on the asymmetry of carotid artery BFV in the hemiparkinsonian NHP model. MPTP infusion was associated with lower BFV in the ipsilateral CCA and resulted in unilateral damage of basal ganglia, whereas higher BFV in the CCA resulted in the collateral circulation of cerebral blood flow. Particularly, the contralateral rotation behavior of the parkinsonian NHP model induced by minimally invasive unilateral injection of MPTP into the carotid artery presented in this study contributes as a preclinical model to evaluate therapeutic strategies for PD, obviating the need for intracerebral parenchymal injection of neurotoxin (Figure 4(b)). In this study, contralateral rotation was observed after apomorphine injection in the unilaterally damaged monkey (C1), with a lower BFV in the ipsilateral CCA. This was presumed to be related to the degree of asymmetrical striatal damage. Apomorphine, a dopamine receptor agonist, is known to act postsynaptically. As a result of hyperstimulation of supersensitive dopamine receptors in the damaged striatum, apomorphine induces rotation contralaterally from the damaged side in hemiparkinsonian rodent and primate models induced by intracerebral injection of 6-hydroxydopamine [44, 45, 51–53]. The quantifiable contralateral rotation behavior correlated with nigrostriatal lesions and was considered the major experimental advantage in estimating the therapeutic and neuroprotective effects of antiparkinsonian pharmacological therapies, compared to the unlesioned side as an internal control [54]. This study may contribute to improving the hemiparkinsonian NHP model. Previous studies using the hemiparkinsonian NHP model have several limitations. First, MPTP infusions were performed in a unidirectional manner in all experimental subjects [1–3, 5, 6]. Based on the presumption that contralateral supply of MPTP via the cerebral collateral circulation is provided by both the CoW and connections of the carotid artery [7, 8], a large sample size was required to obtain monkeys that exhibited hemiparkinsonian signs depending on individual differences in the cerebral collateral circulation. Second, carotid artery incisions require highly detailed surgical techniques and can be fatal. The interventional radiology approach used in this study, from the femoral to the carotid artery, is a traditional method used in stroke studies and is effective and minimally invasive [30, 31, 55]. The interventional radiology method via the femoral artery affords several benefits such as accurate positioning, minimization of risk without carotid artery incisions, and favorable blood patency without external carotid artery ligation. Further, 3R principles (replace, reduce, and refine) should be applied in all animal experiments, and researchers should operate based on the principle of reducing the number of experimental animals used and minimizing their suffering [56]. This study reinforces previous findings that carotid artery blood flow interacts with the collateral circulation of cerebral blood flow [57–60]. The SAIs of FP-CIT BP in subregions of the dorsal striatum were increased in direct proportion to the AIs of CCA BFV in the two damaged monkeys (Supplemental Figure 1). The higher ipsilateral CCA BFV may be related to contralateral striatal damage caused by contralateral blood flow containing MPTP in the collateral circulation. Structural analysis of the A1 segments, which underpin asymmetry in bilateral carotid artery inflow and diameter, provides insight into the contralateral supply of MPTP [16, 20]. The direction of brain blood flow and collateral circulation in the circle of Willis (CoW) are influenced by the diversity of angioarchitecture and vessel diameter [13, 20]. Anatomical variations in the CoW have been reported in previous human studies [13, 57]. Although asymmetry of ACA A1 segments in the CoW was not observed in the three monkeys in this pilot study (Table 1), future studies employing the hemiparkinsonian NHP model should consider asymmetry in the CoW structure to avoid a contralateral supply of MPTP. The ratio of the diameters of the ACA A1 and M1 segments of the middle cerebral artery (MCA) branching from the ICA can be considered important in maintaining collateral circulation [61, 62]. It can be considered that monkeys with a relatively large M1 diameter have an advantage in maintaining ipsilateral flow. In contrast, monkeys with a relatively large A1 diameter tend to maintain contralateral flow. Additionally, although the MPTP may immediately penetrate the ipsilateral and/or contralateral basal ganglia after infusion into the ICA, it is possible that any remaining MPTP, which is not absorbed systemically, ends up in the circulatory system. We conjecture that abnormally high plasma concentrations of RANTES pre-MPTP infusion may underlie the toxicity threshold of MPTP (Figure 4(c)). RANTES is a critical chemokine that regulates the infiltration of T lymphocytes into the basal ganglia in PD [2, 26, 63–66]. Plasma RANTES concentrations were immediately increased after MPTP infusion in all monkeys (Figure 4(c)). In the undamaged monkey, plasma RANTES concentration was abnormally high pre-MPTP infusion. But in the undamaged monkey (C3), plasma RANTES concentration was abnormally high pre-MPTP infusion, and a relatively low increase ranging from plasma concentrations of RANTES pre-MPTP infusion to the high peak of concentrations after infusion of MPTP was observed. Based on this, it is possible that the essential threshold range for damage of the dopaminergic neuronal system caused by MPTP was not exceeded in this monkey. The range increases in the concentration of plasma RANTES induced in MPTP may be more important than the initial concentration. However, in absence of a sham-operated control group to exclude effects of the interventional radiology method, it is difficult to discuss the relevance of changes in plasma RANTES concentrations in the acute phase. Further experiments are needed to establish causality between the plasma RANTES concentrations and the neuronal damage. The limitations of this study are the absence of measurements of CCA BFV before MPTP infusion and the limited number of experimental animals for statistical significance. The effects of MPTP on cerebral BFV have yet to be reported, although studies have examined blood-brain barrier leakage and neurovascular changes induced by MPTP [67–71]. Further studies are warranted to elucidate the changes in cerebral blood flow before and after infusion of MPTP. Additionally, the investigation of hemiparkinsonian behavioral signs using a hand dexterity task for hand function or a gait-sensing walkway system for locomotion should be considered for comparison of MPTP-induced damage to basal ganglia subregions [32, 33].

## 5. Conclusion

Based on our findings, it is suggested that the infusion of MPTP coupled with lower BFV in the ipsilateral CCA and a higher ratio of ipsilateral MCA to ACA may lead to unilateral damage of the basal ganglia. In contrast, higher BFV in the ipsilateral CCA and a lower ratio of ipsilateral MCA to ACA may lead to no or bilateral damage of the basal ganglia due to prominent collateral circulation. The novel strategy of MPTP infusion provides an important conceptual advance of this method for the efficient development of the hemiparkinsonian NHP model (such as a reduction in the number of experimental animals used). The next step for further development of the hemiparkinsonian NHP model is to avoid contralateral supply of MPTP such as artificial and temporary regulation of CCA BFV and/or infusion in the unilateral M1 segment of the MCA using the interventional radiology method. Further, separate bilateral CCA infusions of half-dose MPTP may be useful to induce symmetric parkinsonian signs. It is concluded that asymmetry of blood flow and structural asymmetry of CoW should be taken into consideration in the development of more efficient hemiparkinsonian NHP models.

## Figures and Tables

**Table 1:** Arterial diameter (mm) of the circle of Willis (CoW) segment and carotid arterial segments.

Animals	A1 (mm)		ICA (mm)		CCA (mm)		M1 (mm)	
	Ipsi	Contra	Ipsi	Contra	Ipsi	Contra	Ipsi	Contra
C1	0.5	0.6	1.3	1.4	2.4	2.5	1.1	1.2
C2	1.0	1.0	1.5	1.5	2.0	2.1	1.1	1.1
C3	0.8	0.9	1.1	1.2	1.6	1.6	1.0	0.9

A1, anterior cerebral artery A1 segment; CCA, common carotid artery; ICA, internal carotid artery; M1, middle cerebral artery M1 segment.

## Data Availability

The video clips and datasets used to support the findings of this study are included within the supplementary information files.

## Supplementary Materials

Supplementary Materials Supplemental Figure 1: histogram representing ^18^F-FP-CIT binding potential in the dorsal striatum and subregions of the globus pallidus after left internal carotid artery infusion of MPTP. The dotted line indicates the contralateral dorsal striatum relative to the MPTP-infused side. Binding potential, BP; Contra, Contralateral; Ipsi, Ipsilateral. Supplemental Figure 2: (a) magnetic resonance angiography (MRA) depicting the symmetric structure of the circle of Willis (CoW) in the cerebral arteries of all MPTP-infused cynomolgus monkeys. Supplemental Figure 3: parkinsonian behavior scores were evaluated using the Kurlan scale. Parkinsonian behavior scores were increased after left internal carotid artery infusion of MPTP in unilaterally (C1) and bilaterally (C2) damaged cynomolgus monkeys. Parkinsonian behavior scores of the undamaged cynomolgus monkey (C3) were completely recovered despite an additional infusion of MPTP. ^*∗*^Additional contralateral infusion of MPTP in the undamaged cynomolgus monkey (C3). Supplemental Figure 4: injection of saline was repeated thrice within 28 to 30 weeks, once a week, following MPTP infusion, for comparison with the apomorphine rotation test. The ipsilateral rotations (negative values) were predominantly observed in C1 and C2 monkeys (blue and orange bars) after saline injection. Contrastingly, ipsilateral and contralateral rotations were equally observed in the C3 monkey (gray bar). Plot, mean with standard deviation. Supplemental Video 1: computed tomography angiography (CTA) depicting unilateral cerebral blood flow using a contrast media. Supplemental Video 2: the recorded video of spontaneous behavior before and after 4 weeks of MPTP injections in three monkeys. Supplemental Video 3: the recorded video during the apomorphine rotation test in the unilaterally damaged cynomolgus monkey (C1). Supplemental Table 1: striatal asymmetry index (SAI) calculated using 18F-FP-CIT binding potential.
