# Supplementary material for: Determination of the Unilaterally Damaged Region May Depend on the Asymmetry of Carotid Blood Flow Velocity in Hemiparkinsonian Monkey: A Pilot Study
Source: Parkinsons Dis. 2022 Nov 9;2022:4382145. doi: 10.1155/2022/4382145 (PMC9668443; doi:10.1155/2022/4382145)
Supplement: Supplementary Materials — Supplemental Figure 1: histogram representing 18F-FP-CIT binding potential in the dorsal striatum and subregions of the globus pallidus after left internal carotid artery infusion of MPTP. The dotted line indicates the contralateral dorsal striatum relative to the MPTP-infused side. Binding potential, BP; Contra, Contralateral; Ipsi, Ipsilateral. Supplemental Figure 2: (a) magnetic resonance angiography (MRA) depicting the symmetric structure of the circle of Willis (CoW) in the cerebral arteries of all MPTP-infused cynomolgus monkeys. Supplemental Figure 3: parkinsonian behavior scores were evaluated using the Kurlan scale. Parkinsonian behavior scores were increased after left internal carotid artery infusion of MPTP in unilaterally (C1) and bilaterally (C2) damaged cynomolgus monkeys. Parkinsonian behavior scores of the undamaged cynomolgus monkey (C3) were completely recovered despite an additional infusion of MPTP. ∗Additional contralateral infusion of MPTP in the undamaged cynomolgus monkey (C3). Supplemental Figure 4: injection of saline was repeated thrice within 28 to 30 weeks, once a week, following MPTP infusion, for comparison with the apomorphine rotation test. The ipsilateral rotations (negative values) were predominantly observed in C1 and C2 monkeys (blue and orange bars) after saline injection. Contrastingly, ipsilateral and contralateral rotations were equally observed in the C3 monkey (gray bar). Plot, mean with standard deviation. Supplemental Video 1: computed tomography angiography (CTA) depicting unilateral cerebral blood flow using a contrast media. Supplemental Video 2: the recorded video of spontaneous behavior before and after 4 weeks of MPTP injections in three monkeys. Supplemental Video 3: the recorded video during the apomorphine rotation test in the unilaterally damaged cynomolgus monkey (C1). Supplemental Table 1: striatal asymmetry index (SAI) calculated using 18F-FP-CIT binding potential. [file 4382145.f1.zip › Suppemental materials.pdf]

Supplementary Materials

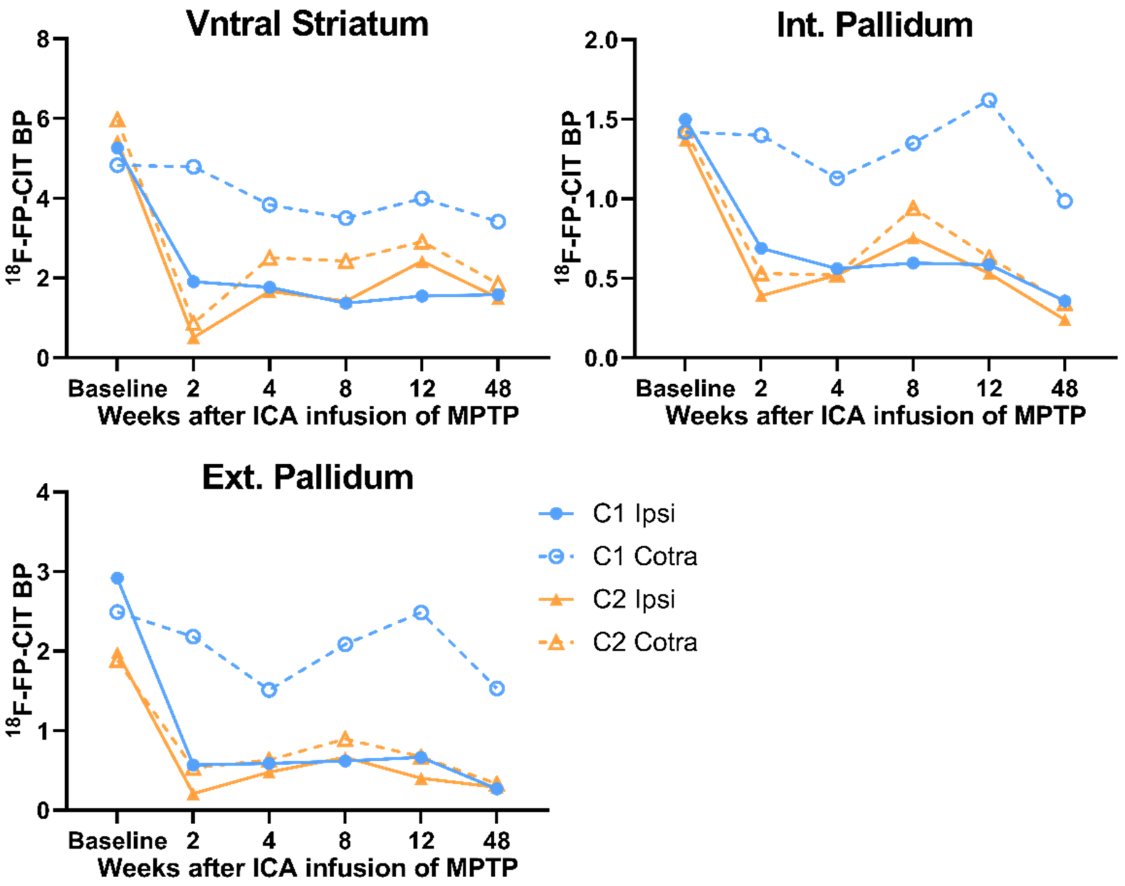

Supplemental Fig 1: Histogram representing ^{18}F-FP-CIT binding potential in the dorsal striatum and sub-regions of the globus pallidus after left internal carotid artery infusion of MPTP. The dotted line indicates the contralateral dorsal striatum relative to the MPTP-infused side. Binding potential, BP; Contra, Contralateral; Ipsi, Ipsilateral.

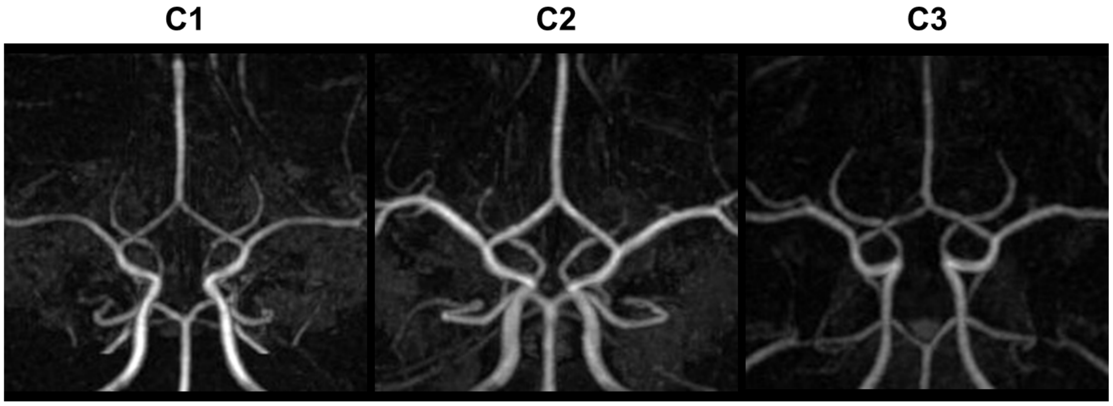

Supplemental Fig 2: (A) Magnetic resonance angiography (MRA) depicting the symmetric structure of the circle of Willis (CoW) in the cerebral arteries of all MPTP-infused cynomolgus monkeys.

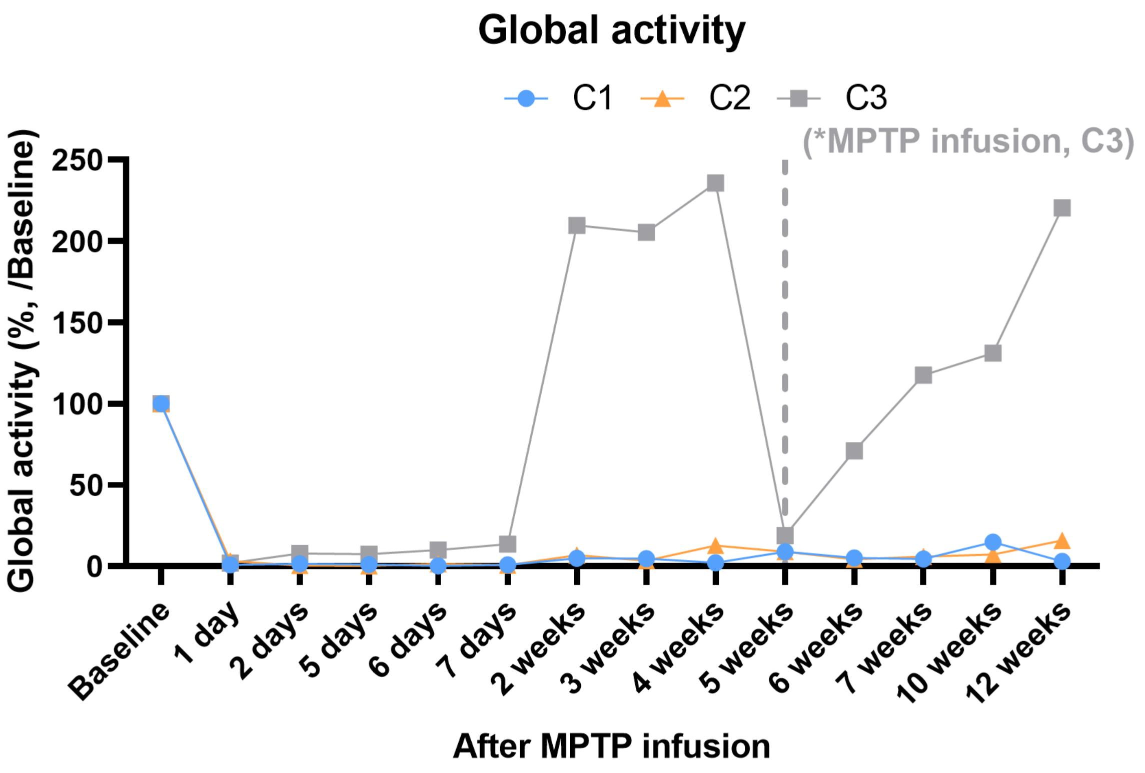

Supplemental Fig 3: Parkinsonian behavior scores were evaluated using the Kurlan scale. Parkinsonian behavior scores were increased after left internal carotid artery infusion of MPTP in unilaterally (C1) and bilaterally (C2) damaged cynomolgus monkeys. Parkinsonian behavior scores of the undamaged cynomolgus monkey (C3) was completely recovered despite additional infusion of MPTP. *Additional contralateral infusion of MPTP in the undamaged cynomolgus monkey (C3).

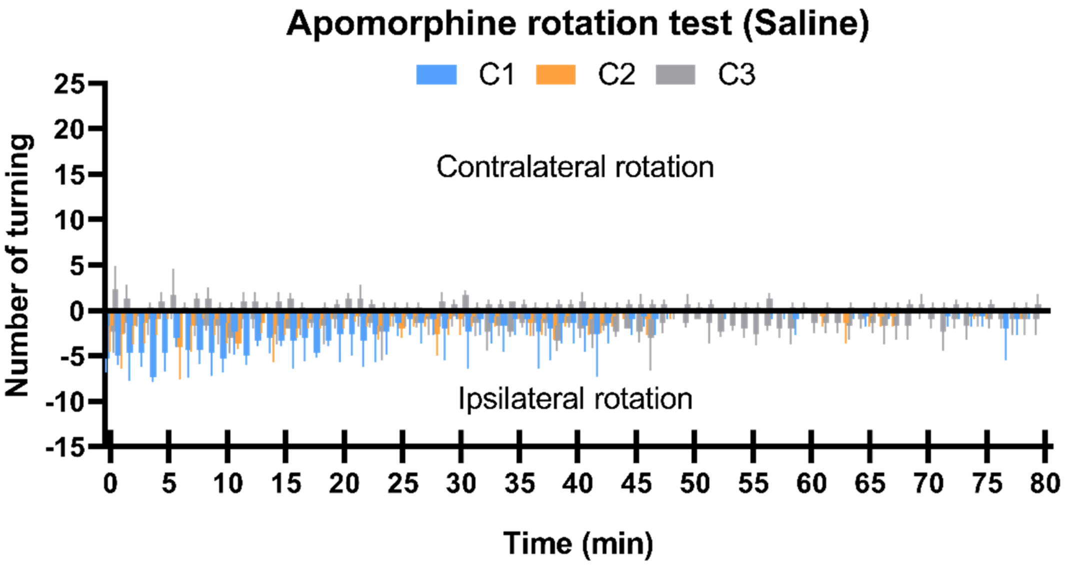

Supplemental Fig 4: Injection of saline was repeated thrice within 28 to 30 weeks, once a week, following MPTP infusion, for comparison with apomorphine rotation test. The ipsilateral rotations (negative values) were predominantly observed in C1 and C2 monkey (blue and orange bar) after saline injection. Contrastingly, ipsilateral and contralateral rotations were equally observed in the C3 monkey (gray bar). Plot, mean with standard deviation.

Suppemental
Video 1.avi

Supplemental Video 1: Computed tomography angiography (CTA) depicting unilateral cerebral blood flow using a contrast media.

Suppemental
Video 2 (for review

Supplemental Video 2: The recorded video during spontaneous behavior before and after 4 weeks of MPTP injections in three monkeys.

Suppemental
Video 3 (for review

Supplemental Video 3: The recorded video during apomorphine rotation test in the unilaterally damaged cynomolgus monkey (C1).

Please download the links of videos in the Google drive below.
https://drive.google.com/drive/folders/1giLu253rgN_-q8JEvnQEih_YM3FsLosa?usp=sharing

Supplemental Table 1: Striatal asymmetry index (SAI) calculated using ^{18}F-FP-CIT binding potential

	Before (a)		8 weeks after MPTP (b)		(b-a)
	Mean	SD	Mean	SD	
C1	0.059	0.036	1.478	0.074	1.419
C2	0.035	0.034	0.964	0.221	0.930
C3	0.045	0.051	0.038	0.033	0.007
IM-MPTP (n=5)	0.154	0.115	0.190	0.175	0.036
